# Liraglutide ameliorates diabetic-induced testicular dysfunction in male rats: role of GLP-1/Kiss1/GnRH and TGF-β/Smad signaling pathways

**DOI:** 10.3389/fphar.2023.1224985

**Published:** 2023-07-11

**Authors:** Maha Abdelhamid Fathy, Amira Ebrahim Alsemeh, Marwa A. Habib, Hanim M. Abdel-nour, Doaa M. Hendawy, Asmaa Monir Eltaweel, Adel Abdelkhalek, Mona M. Ahmed, Maha K. Desouky, Jinlian Hua, Liana Mihaela Fericean, Ioan Banatean-Dunea, Ahmed Hamed Arisha, Tarek Khamis

**Affiliations:** ^1^ Medical Physiology Department, Faculty of Medicine, Zagazig University, Zagazig, Egypt; ^2^ Human Anatomy and Embryology Department, Faculty of Medicine, Zagazig University, Zagazig, Egypt; ^3^ Medical Biochemistry Department, Faculty of Medicine, Zagazig University, Zagazig, Egypt; ^4^ Basic Medical Science Department of Anatomy and Embryology, College of Medicine-King Saud Abdulaziz, University for Health Sciences—Kingdom of Saudi Arabia, Jeddah, Saudi Arabia; ^5^ Human Anatomy and Embryology Department, Faculty of Medicine, Zagazig University, Zagazig, Egypt; ^6^ Faculty of Veterinary Medicine, Badr University in Cairo, Badr, Egypt; ^7^ Department of Forensic Medicine and Toxicology, Faculty of Veterinary Medicine, Zagazig University, Zagazig, Egypt; ^8^ Department of Anatomy, Faculty of Medicine, Minia University, Minia, Egypt; ^9^ College of Veterinary Medicine/Shaanxi Centre of Stem Cells Engineering and Technology, Northwest Agriculture and Forestry University, Yangling, Shaanxi, China; ^10^ Department of Biology, Faculty of Agriculture, University of Life Sciences, King Mihai I” from Timisoara [ULST], Timisoara, Romania; ^11^ Department of Animal Physiology and Biochemistry, Faculty of Veterinary Medicine, Badr University in Cairo, Badr, Egypt; ^12^ Department of Physiology and Laboratory of Biotechnology, Faculty of Veterinary Medicine, Zagazig University, Zagazig, Egypt; ^13^ Department of Pharmacology and Laboratory of Biotechnology, Faculty of Veterinary Medicine, Zagazig University, Zagazig, Egypt

**Keywords:** liraglutide, GLP-1, kisspeptin, HPG axis, antioxidant enzymes, steroidogenesis, TGF-β 1

## Abstract

**Introduction:** Glucagon-like peptide -1 (GLP-1) is released by intestinal cells to stimulate glucose-dependent insulin release from the pancreas. GLP-1 has been linked to ameliorating obesity and/or diabetic complications as well as controlling reproductive function. Liraglutide is a GLP-1 receptor agonist (GLP-1RA) with 97% homology with GLP-1. The main objective of this study was to investigate the ameliorative role of liraglutide in diabetic-induced reproductive dysfunction in male rats.

**Methods:** Rats were randomly allocated into 3 groups; a control group, a diabetic group, and a liraglutide-treated diabetic group.

**Results:** In the diabetic group, a significant increase in BMI, FBG, HbA1c, HOMA-IR, TC, TAG, LDL, IL6, TNFα, and MDA, as well as decreased serum insulin, HDL, GSH, total testosterone, LH, and FSH, were shown compared to the control group. Furthermore, A significant downregulation in relative hypothalamic gene expression of GLP-1R, PPAR-α, PGC-1α, kiss, kiss1R, leptin, leptin R, GnRH GLP-1R, testicular PGC-1α, PPARα, kiss1, kiss1R, STAR, CYP17A1, HSD17B3, CYP19A, CYP11A1, and Smad7, as well as upregulation in hypothalamic GnIH and testicular TGF- β and Smad2 expression, were noticed compared to the control group. Liraglutide treatment significantly improved such functional and structural reproductive disturbance in diabetic rats.

**Conclusion:** GLP-1RAs ameliorated the deleterious effects of diabetes on reproductive function by targeting GLP-1/leptin/kiss1/GnRH, steroidogenesis, and TGF- β/Smad pathways.

## 1 Introduction

Type II diabetes is associated with impaired glycemic metabolism which affects different physiologic functions including testicular activity and male fertility mostly due to vascular damage leading to reduced blood flow and oxygenation to the testes, oxidative stress by producing free radicals that affect sperm quality by damaging DNA and reducing motility, neuropathy causing erectile dysfunction or retrograde ejaculation and nephropathy leading to hormonal imbalances that affect sperm production (Viigimaa et al., 2020; He et al., 2021). The diabetic-induced pathogenesis also involves provoking chronic inflammation and mitochondrial damage via long-term hyperglycemia and insulin resistance which affect the male reproductive system at different levels; centrally via impairment of hypothalamic–pituitary–gonadal axis (HPG) and locally through induction of structural injuries of reproductive organs including testicular atrophy, seminiferous tubule destruction, stromal and spermatogenic cell damage (Maresch et al., 2018). In humans, it was shown that almost half of the young obese and diabetic men have impaired gonadal function (Dhindsa et al., 2018).

Type II diabetes leads to impaired metabolic and sex hormone production, transport, and effects (Gambineri and Pelusi, 2019). In addition to the induction of insulin resistance, Type II diabetes can also impair other metabolic hormones such as leptin and adiponectin (Al-Suhaimi and Shehzad, 2013) as well as reduce steroidogenesis and testosterone levels (Nna et al., 2019). GLP-1, an incretin, is a postprandial hormone produced by intestinal L cells in the distal ileum and colon (Kreymann et al., 1987). It has several metabolic effects as it stimulates glucose-dependent insulin secretion from β-cells of the pancreas, inhibits glucagon secretion, slows gastric emptying, and decreases food intake leading to a reduction of weight (Gutzwiller et al., 1999). GLP-1 receptors are expressed also in extra-pancreatic tissue including muscle, adipose tissue, the hypothalamus, and the pituitary gland (Oride et al., 2017). GLP-1 receptor (GLP-1R) expression was reported in rodent and human testis in both *in vitro* and *in vivo* studies, thus implementing potential regulatory effects on male reproductive function (Caltabiano et al., 2020; Cannarella et al., 2021). Yet, such effects remain not fully clear or even controversial. GLP-1 receptor agonists (GLP-1RAs) are currently used to ameliorate obesity and type II diabetes. GLP-1RAs include liraglutide with 97% homology to GLP-1 and longer half-life (Barrea et al., 2020) as well as exendin-4 with 53% homology to GLP-1 (Zhou et al., 2009). *In-vitro* administration of GLP-1 analog to neuronal cell line induced dose-dependent increase in LH (Beak et al., 1998). Moreover, while acute treatment with GLP-1 increased pre-ovulatory LH surge in female rats (Outeiriño-Iglesias et al., 2015), chronic use of GLP-1RA agonist reduced LH mainly by affecting the kisspeptin/GnRH hypothalamic system (Bednarz et al., 2022). GLP-1 receptor knockout mice showed reduced seminal vesicle and testicular weight as well as delayed onset of puberty (MacLusky et al., 2000). In human studies, liraglutide treatment improved metabolic and reproductive function and corrected the suppression in the HPG axis (Giagulli et al., 2015; Jensterle et al., 2019). However, other reports indicated a reduction in testosterone pulse and a prolongation of pulse duration after liraglutide infusion with no effect on mean LH or testosterone (Jeibmann et al., 2005). Another study reported no effect on testosterone or LH after GLP-1 infusion (Izzi-Engbeaya et al., 2020).

Transforming growth factor β (TGF-β) is an important regulator of cell proliferation and differentiation (Massagué and Wotton, 2000). TGF-β is recognized as a pro-fibrotic and pro-inflammatory factor linked to several disorders such as cancer and autoimmune fibrosis (Zhang et al., 2012) and is involved in the control of reproductive function (Itman et al., 2006; Kabel, 2018) as it contributes to the progression of reproductive dysfunction mostly by stimulating testicular fibroblasts and sperm apoptosis (Salama et al., 2001; Kabel, 2018). TGF- β receptors trigger its action through phosphorylation of R-Smads (Smad1, Smad2, Smad3, Smad5, and Smad8) (Blobe et al., 2000) inducing the transduction of downstream gene transcription (ten Dijke and Hill, 2004; Tzavlaki and Moustakas, 2020). I-Smads (Smad 6 and Smad 7) are inhibitory proteins that antagonize the activity of R-Smads (Hayashi et al., 1997; Imamura et al., 1997). Upregulation of the TGF- β/Smad pathway was found in diabetic rats and was associated with pulmonary (Zhao et al., 2014), renal (Chen et al., 2018), and testicular fibrotic damage (Liu et al., 2019). Liraglutide treatment decreased TGF-β and immune cell infiltration in pulmonary fibrosis (Gou et al., 2014; Zhao et al., 2014). Liraglutide and exendin-4 treatment attenuated TGF-β expression in the renal cortex and decreased inflammation and oxidative stress in diabetic nephropathy (Sancar-Bas et al., 2015; Chen et al., 2018). Yet, the effect of GLP-1RAs on the testicular TGF- β/Smad pathway in diabetic rats remains not fully elucidated. Overall, this study was designed to investigate the effects of GLP-1RA, liraglutide, on the HPG axis and testicular function in diabetic rats as well as the possible involvement of antioxidant defenses, GLP-1/leptin/kisspeptin/GnRH and TGF-β/Smad pathways.

## 2 Materials and methods

### 2.1 Animals and experimental design

24 adult male albino rats were acquired from the animal house, faculty of Veterinary Medicine-Zagazig University. The rats aged 8–10 weeks with an average weight of 180–200 g. Rats received standard laboratory animal care throughout the study period and the study protocol was approved by Zagazig University-institutional committee of animal care and use (approval number: ZU-IACUC/3/F/377/2022). After acclimatization for 1 week, rats were randomly allocated into three equal groups; Group I: control group was given a standard chew diet (10% fat, 20% protein, and 70% carbohydrate; 3.85 kcal/g) (Tian et al., 2020), and after 8 weeks, received a single intraperitoneal injection of the vehicle (sodium citrate 0.1 mol/L). Group II: the diabetic group was fed a high-fat diet (45% fat, 20% protein, and 35% carbohydrate; 4.73 kcal/g) (Tian et al., 2020), after 8 weeks, diabetes was induced by a single intra-peritoneal injection with streptozotocin (STZ) at a dose of 25 mg/kg in 0.1 mol/L sodium citrate, ph 4.5. Group III: diabetic + liraglutide, the same protocol (HFD + STZ) for induction of diabetes as group II, then rats received liraglutide (0.4 mg/kg/day) subcutaneous injection for 8 weeks (Zhang et al., 2018; Sukumaran et al., 2020). Rats were given glucose solutions of 10% for 48 h orally six hours after the STZ injection to avoid hypoglycemia (Hou et al., 2012). The blood glucose of the rat tail vein was checked using a One Touch Glucometer. Rats with fasting blood glucose (FBG) ≥ 200 mg/dL in two consecutive analyses were considered type 2 diabetic (Hou et al., 2012). Control and diabetic groups were injected with the same volume of normal saline.

### 2.2 Body weight change, anthropometric measures, and blood sampling

Body weight change was measured weekly. BMI was measured at the start and the end of the study = body mass (g)/length (cm^2^); the cut-off for obesity BMI >0.68 g/cm^2^ (Novelli et al., 2007). At the end of the experiment, following overnight fasting, rats were anesthetized using ketamine (100 mg/kg i. p). Blood samples were taken from retro-orbital plexus either in tubes with 3.2% sodium citrate solution then centrifuged for 10 min at 3,000 r.p.m to separate plasma or without anticoagulant then allowed to clot, centrifuged for 20 min at 3,000 r.p.m. to separate serum. Such samples were then kept at −20°C.

### 2.3 Tissue collection and preparation of testicular homogenate

After blood samples were taken, rats were killed by decapitation. The hypothalamus, pituitary, and 30 mg of testicular tissue were snap-frozen and stored in liquid nitrogen for gene expression. The other testis was divided into two parts, one part was sliced, then homogenized in Phosphate buffer saline (PBS), centrifuged for 10 min at 1,000 r.p.m and the supernatant was separated and used for oxidative stress marker assay. The other part of the testis was placed in 10% neutral buffer formalin for the histopathological and immunohistochemical examination.

### 2.4 Assessment of biochemical parameters

Enzymatic colorimetry was done using an automated biochemistry analyzer a Cobas^®^ 6,000 analyzer (Roche Diagnostics Ltd., Switzerland) to measure; Fasting blood glucose (FBG) using glucose enzymatic (GOD-PAP)—Kits, glycohemoglobin (HbA1c), Serum lipid profile. HOMA-IR (homeostatic model assessment of insulin resistance) was calculated: HOMA-IR = fasting serum glucose (mg/dL) x fasting serum insulin (µIU/mL)/405 (Matthews et al., 1985).

### 2.5 Hormonal assay

Serum insulin was determined using a rat insulin ELISA kit (EMD Millipore Corporation, United States). Total serum testosterone, follicle-stimulating hormone (FSH), and luteinizing hormone (LH) were measured using a rat ELISA kit (Roche Diagnostics, USA) by applying the manufacturer’s guidelines. The absorbance of colorimetric solutions was assayed using a Utrao microplate reader (Shanghai, China).

### 2.6 Inflammatory and oxidative stress markers

IL-6 and TNF-α in testicular tissues were evaluated using commercial kits (Sigma-Aldrich, Germany). Malondialdehyde (MDA) and reduced glutathione (GSH) in testicular homogenate were calorimetrically measured by a commercially available kit (Biodiagnostic, Giza, Egypt) following manufacturer’s protocol using a spectrophotometer (Spectronic 3,000 Array, Germany).

### 2.7 RT-qPCR

Total RNA was extracted from the hypothalamic, pituitary, and testicular tissue using Qiazol (Qiagen; Hilden, Germany) according to the manufacturer’s guidelines. To estimate the total RNA concentration, we used a NanoDrop^®^ ND–1000 Spectrophotometer (NanoDrop Technologies; Wilmington, Delaware, United States). Using the cDNA Reverse Transcription Kit with High-Capacity (Applied Biosystems™, United States), reverse transcription of total RNA to cDNA was performed. Amplification of cDNA was conducted in a real-time thermal cycler Rotor-Gene Q (Qiagen, Germany) (Khamis et al., 2020; Khamis et al., 2021) with a SYBER Green master mix, TOPreal™ qPCR 2 × PreMIX (Enzynomics, Korea) with oligo-NTPs primers (Sangon Biotech, Beijing, China) mentioned in Table 1. The relative gene expression over the normalizer gene Gapdh was finally calculated and represented as a percentage from the control and the fold change was estimated as 2^−ΔΔCT^ (Schmittgen and Livak, 2008).

**TABLE 1:** Primers sequences of targeted genes.

Gene	Forward primer sequence (5′to 3′)	Reverse primer sequence (5′to 3′)	Product size	Accession no.
CYP11A1	AAGTATCCGTGATGTGGG	TCA​TAC​AGT​GTC​GCC​TTT​TCT	127	NM_017286.3
CYP17A1	TGG​CTT​TCC​TGG​TGC​ACA​ATC	TGA​AAG​TTG​GTG​TTC​GGC​TGA​AG	90	NM_012753.2
CYP19A1	GCT​GAG​AGA​CGT​GGA​GAC​CTG	CTC​TGT​CAC​CAA​CAA​CAG​TGT​GG	178	NM_017085.2
Gapdh	GCA​TCT​TCT​TGT​GCA​GTG​CC	GGT​AAC​CAG​GCG​TCC​GAT​AC	91	NM_017008.4
GLP-1R	CTT​GGA​GAC​ATA​GAA​GGG​GGA​C	AGG​AGC​ATG​CCT​CTG​GGT​AG	128	NM_172091.2
GnIH	AGA​GCA​ACC​TAG​GAA​ACG​GGT​GTT	AGG​ACT​GGC​TGG​AGG​TTT​CCT​ATT	84	NM_023952.1
GnRH1	AGG​AGC​TCT​GGA​ACG​TCT​GAT	AGC​GTC​AAT​GTC​ACA​CTC​GG	100	NM_012767.2
GnRHr	TCA​GGA​CCC​ACG​CAA​ACT​AC	CTG​GCT​CTG​ACA​CCC​TGT​TT	182	NM_031038.3
HSD17B3	AGT​GTG​TGA​GGT​TCT​CCC​GGT​ACC​T	TAC​AAC​ATT​GAG​TCC​ATG​TCT​GGC​CAG	161	NM_054007.1
Kiss-1	TGC​TGC​TTC​TCC​TCT​GTG​TGG	ATT​AAC​GAG​TTC​CTG​GGG​TCC	110	NM_181692.1
Kiss-1r	CTT​TCC​TTC​TGT​GCT​GCG​TA	CCT​GCT​GGA​TGT​AGT​TGA​CG	102	NM_023992.1
Leptin	CAA​TGA​CAT​TTC​ACA​CAC​GCA​G	AGA​TGG​AGG​AGG​TCT​CGC​AG	204	NM_013076
Leptinr	GTG​TCC​TTC​CTG​ACT​CCG​TAG	GTT​ATT​CTC​TGG​AAA​GAC​TGG​CT	119	NM_012596
PGC1α	TTC​AGG​AGC​TGG​ATG​GCT​TG	GGG​CAG​CAC​ACT​CTA​TGT​CA	70	NM_031347.1
Pparα	GTC​CTC​TGG​TTG​TCC​CCT​TG	GTC​AGT​TCA​CAG​GGA​AGG​CA	176	NM_013196.2
Smad-2	CAA​ACG​TGC​ACA​GGT​GAC​AG	GAC​TGG​CGT​TGG​AAG​AAG​GA	83	NM_001277450.1
Smad-7	GAG​TCT​CGG​AGG​AAG​AGG​CT	CTG​CTC​GCA​TAA​GCT​GCT​GG	84	NM_030858.2
StAr	CCC​AAA​TGT​CAA​GGA​AAT​CA	AGGCATCTCCCCAAAGTG	187	NM_031558.3
TGF-β1	AGG​GCT​ACC​ATG​CCA​ACT​TC	CCA​CGT​AGT​AGA​CGA​TGG​GC	168	NM_021578.2

### 2.8 Histological evaluation by H&E stain

Fixed testicular samples were dehydrated, cleared, and embedded in paraffin. Serial 5 μm sections of the testis were stained with hematoxylin and eosin (H&E) to evaluate the histological architecture changes in the testis tissues (Layton and Suvarna, 2013).

### 2.9 Immunohistochemical analysis

Paraffin-prepared sections were stained using the avidin–biotin peroxidase system for the Kisspeptin, androgen receptor, TNFα, Nrf2, and Ki67 detection using a primary antibody (rabbit polyclonal antibody). Following three PBS washes (5 min each), the sections were incubated for 30 min at room temperature with biotinylated secondary antibody and avidin–biotin complex (Vectastain^®^ ABC-peroxidase kit, Vector Laboratories, Burlingame, CA). The color was developed using 3,3′ -diaminobenzidine (DAB) substrate (Vector^®^ DAB, Vector Laboratories). Mayer’s hematoxylin was used as a counterstain. The stained sections were examined by light microscopy (LEICA ICC50 W) in the Image Analysis Unit of the Human Anatomy and Embryology Department, Zagazig University (Khamis et al., 2023).

### 2.10 Morphometric study

The mean number of positive immune reactivity for Kisspeptin, androgen receptors, TNFα, Nrf2, and Ki67 in the tubular epithelium/high power field were measured in five non-overlapping fields from five different sections of different rats in each group. Measurements were performed at the image-analyzing unit presented in the Anatomy Department, Faculty of Medicine, Zagazig University using the image analyzer computer system, (Leica Qwin 500, Microsystems Imaging Solutions Ltd., Cambridge, United Kingdom).

### 2.11 Statistical analysis

Data were expressed as mean ± S.E.M. GraphPad Prism^®^, Version 9.2 was used for processing statistical analysis (GraphPad Software Inc., San Diego, California, United States). All data were normally distributed and were analyzed by ANOVA test to compare means followed by Tukey–Kramer multiple comparisons tests. *p*-value <0.05 was considered significant.

## 3 Results

### 3.1 Biochemical parameters

**FIGURE 1:**
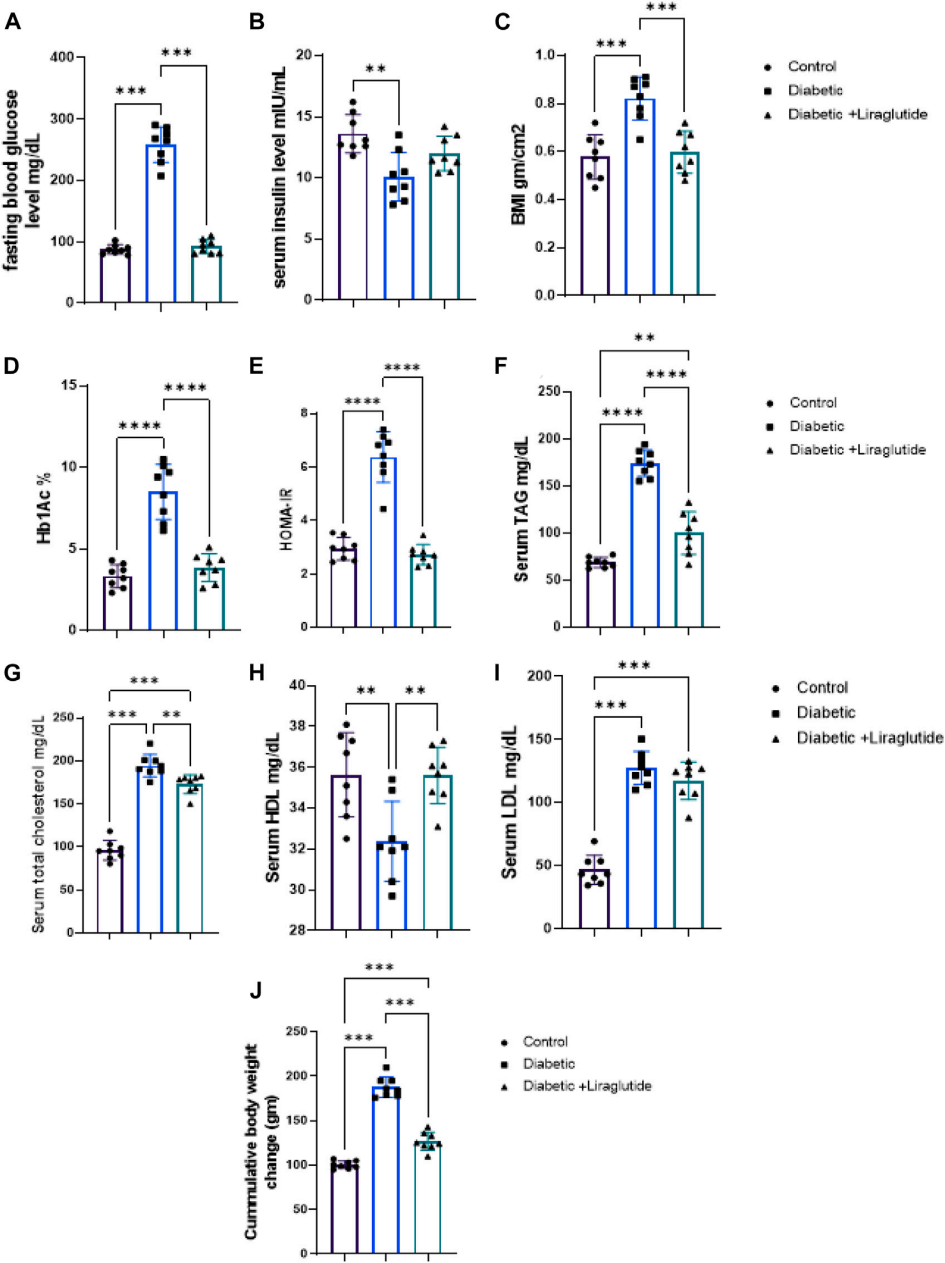
Effect of liraglutide on Biochemical parameters: **(A–I)**. **(A)** FBG, **(B)** serum insulin, **(C)** BMI, **(D)** HbA1c, **(E)** HOMA-IR, **(F)** TAG, **(G)** TC, **(H)** HDL, **(I)** LDL and **(J)** Cumulative body weight change. Data are expressed as means ± SEM. N = 8. *, **, ***, **** indicate significant difference (*p* < 0.05, *p* < 0.01, and *p* < 0.001).

A significant increase in B. wt, BMI, and glucose homeostatic parameters; FBG, HbA1c, HOMA-IR (*p* < 0.0001), and decreased serum insulin (*p* < 0.01) were noticed in the diabetic group compared to the control in Figures 1A–E, J. Lipid profile parameters (TC, TAG, and LDL-c) significantly increased (*p* < 0.0001) while HDL significantly decreased (*p* < 0.01) *versus* control in Figures 1F–I. Liraglutide treatment induced a significant decrease in B.wt, BMI (*p* < 0.001), FBG, HbA1c, HOMA-IR, TAG, (*p* < 0.0001), and TC (*p* < 0.01), meanwhile, HDL significantly increased (*p* < 0.01) compared to the diabetic group in Figures 1A–J.

### 3.2 Inflammatory mediators and oxidative stress markers

**FIGURE 2:**
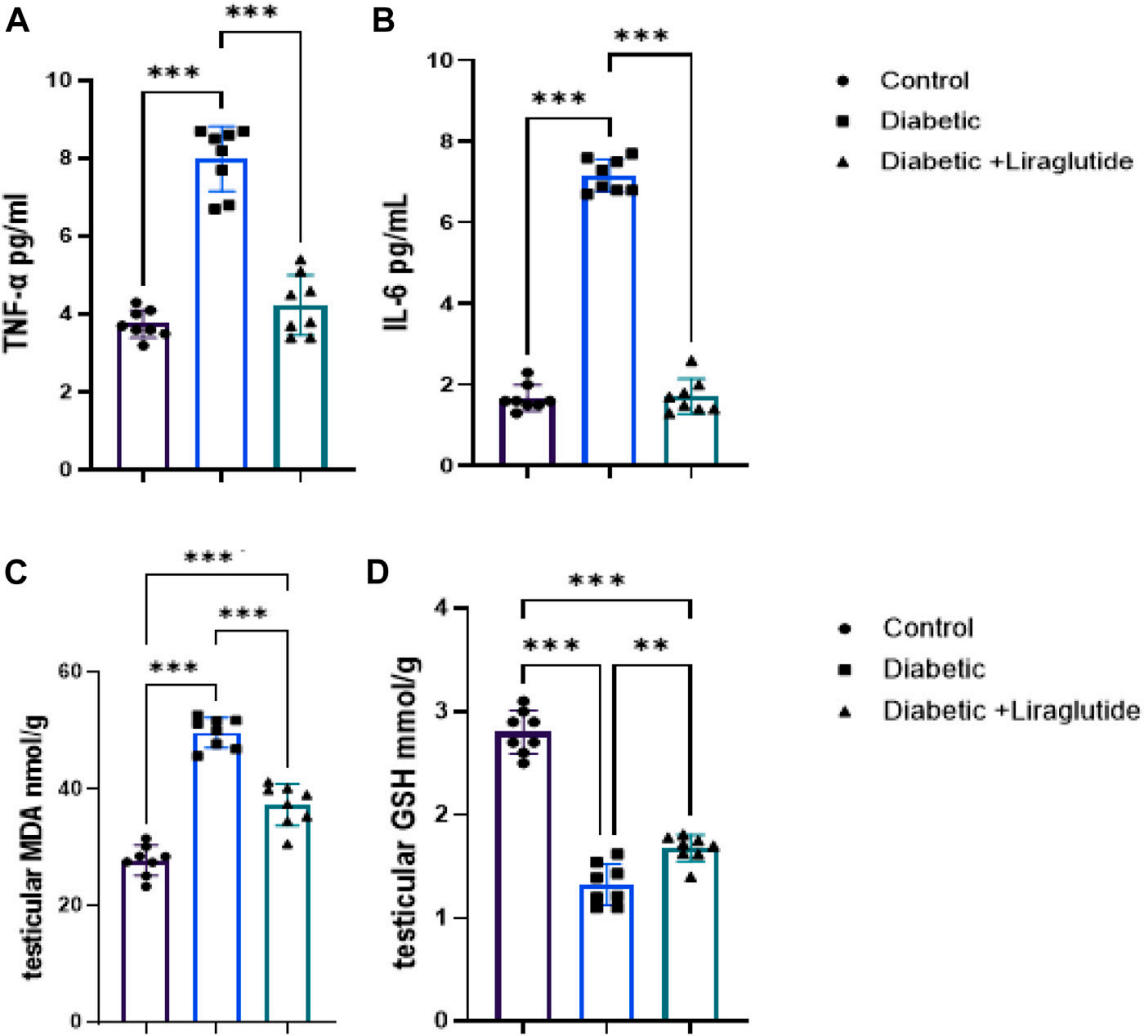
Effect of liraglutide on Inflammatory mediators and oxidative stress markers: **(A–D)**. **(A)** TNFα, **(B)** IL6, **(C)** MDA, and **(D)** GSH. Data are expressed as means ± SEM. N = 8. *, **, *** indicate significant difference (*p* < 0.05, *p* < 0.01, and *p* < 0.001).

A significant increase in inflammatory mediators (IL6 and TNFα) and the lipid peroxidation marker, MDA, was found in group II *versus* control (*p* < 0.0001) in Figures 2A–C, meanwhile, the antioxidant enzyme, GSH, significantly decreased in group II *versus* control (*p* < 0.0001) in Figure 2D. In group III, a significant decrease in IL6, TNFα and MDA levels (*p* < 0.0001) and a significant increase in GSH (*p* < 0.0001) were observed after the liraglutide treatment compared with the diabetic group in Figures 2A–D.

### 3.3 Hormonal profile

**FIGURE 3:**
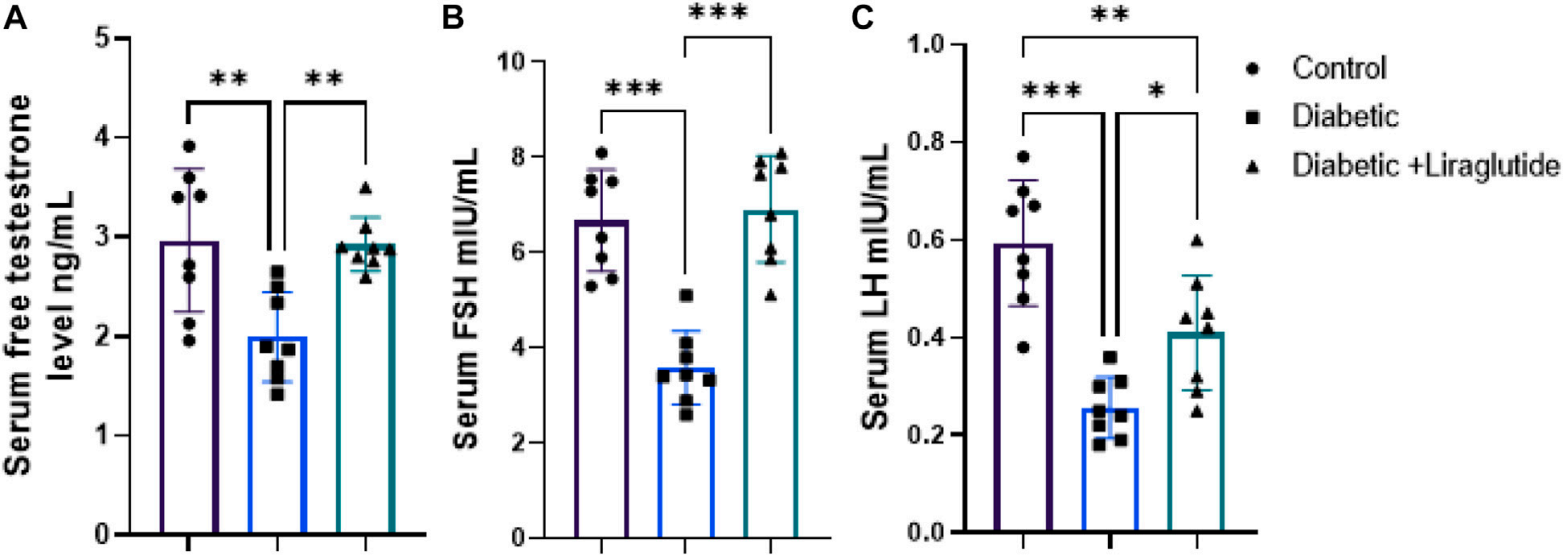
Effect of liraglutide on Hormonal profile: **(A–C)**. **(A)** total testosterone, **(B)** FSH, and **(C)** LH. Data are expressed as means ± SEM. N = 8. *, **, *** indicate significant difference (*p* < 0.05, *p* < 0.01, and *p* < 0.001). and 3.4. H&E stain histological results.

A significant decrease in total testosterone (*p* < 0.01), LH, and FSH (*p* < 0.0001) were observed in group II *versus* control, meanwhile, liraglutide treatment in group III significantly increased their level compared to the diabetic group (*p* < 0.01, *p* < 0.05 and *p* < 0.0001 respectively) in Figures 3A–C.

**FIGURE 4:**
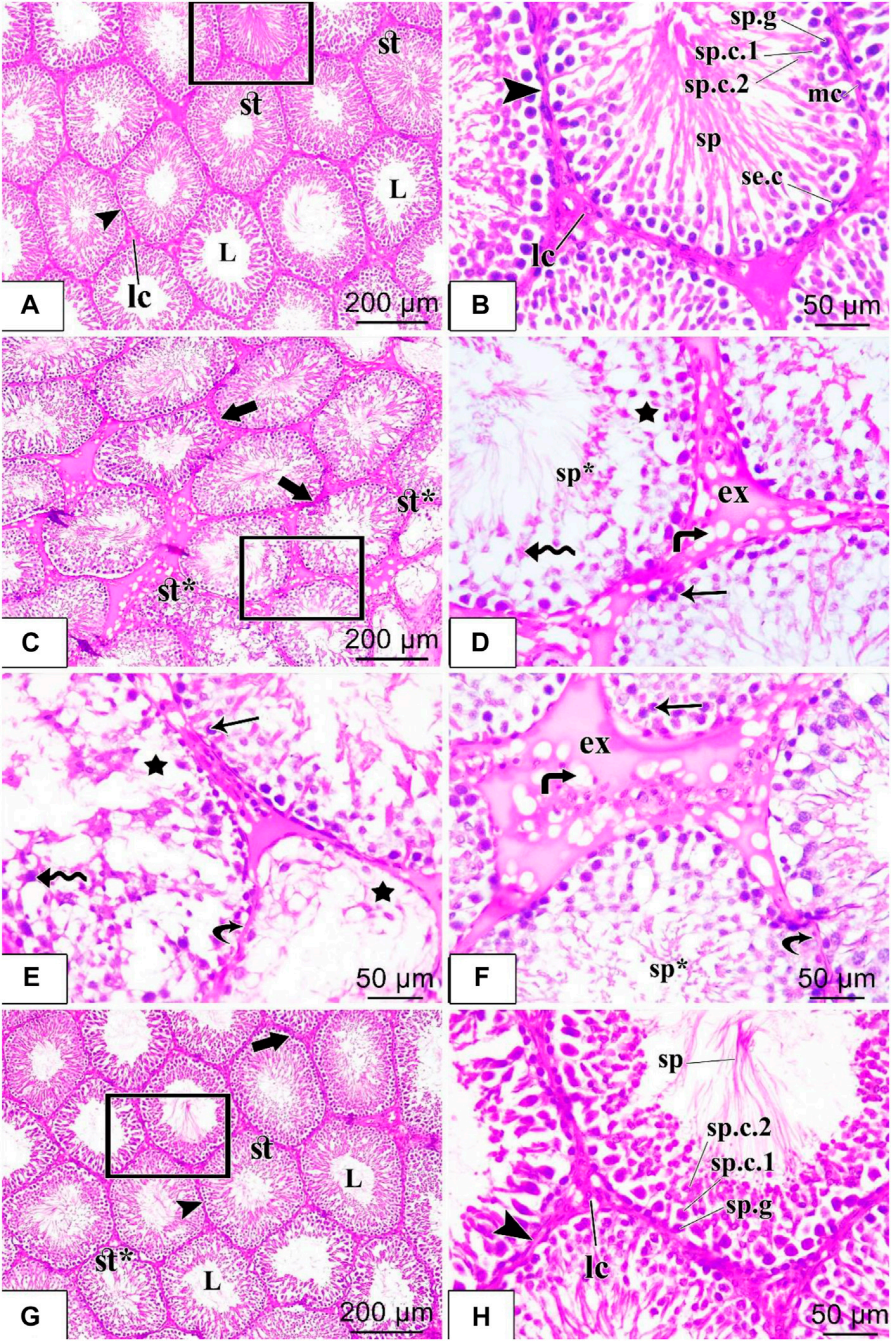
H&E staining of testicular tissues of control, STZ rats, and STZ rats treated with liraglutide **(A–H)**. **(A)** A stained section in the control group: shows closely packed seminiferous tubules (st) which are lined by spermatogenic epithelium and have patent lumina (L). The tubules are separated from each other by interstitial connective tissue (arrowhead) containing the Leydig cells (Ic); **(B)** stained section in the control group: higher magnification of the squared area of the figure (a) shows different stages of spermatogenic cells in the form of spermatogonia (sp.g), primary spermatocytes (sp.c.1), secondary spermatocytes (sp.c.2), spermatids and sperms (sp). Sertoli cells with their large pale nuclei were noticed in between (se.c). The tubules were ensheathed by a well-defined basement membrane and connective tissue (arrowhead) containing flattened myoid cells with flattened nuclei (mc). Clusters of Leydig cells (Ic) are seen in the interstitial tissue; **(C)** Stained section in the diabetic group: shows irregular contour (thick arrow) of seminiferous tubules, vacuolated germinal epithelial cells (star), and damaged, disorganized tubules (st*); **(D)** Stained section in the diabetic group: higher magnification of the squared area of figure **(C)** shows homogenous eosinophilic exudate (ex) with vacuolation (bent arrow) in the interstitium. Large empty spaces are seen between spermatogenic cells (star), which appear disorganized with detached cells (zigzag arrow) and pyknotic nuclei (arrow). Sperm flagella are hardly noticed in the lumens of seminiferous tubules (sp*); **(E,F)** Other fields of rat testicular tissue sections in the diabetic group: show parts of the tubules with thin detached basal lamina (curved arrow) and depleted of most of the spermatogenic cells with large empty spaces in between the cells (star) are seen. Many germ cells are detached (zigzag arrow), and shrunken with pyknotic nuclei (arrow). Sperm flagella are hardly noticed in the lumen of seminiferous tubules (sp*). Large homogenous eosinophilic exudate (ex) with vacuolation (bent arrow) is seen in the interstitium; **(G)** Stained section in diabetic + liraglutide group: shows most of the seminiferous tubules (st) restore the normal architecture, their regular wall (arrowhead) is formed of nearly normally arranged germinal epithelium. Most of their lumina (L) contained aggregation of sperms, and a few seminiferous tubules were still disorganized and damaged (st*). Interstitium in-between the tubules show normal width except for little homogenous vacuolated eosinophilic material between a few tubules (thick arrow); **(H)** Stained section in diabetic + liraglutide group: higher magnification of the squared area of figure **(G)** exhibits different stages of spermatogenic cells resting on regular basal lamina (arrowhead), in the form of spermatogonia (sp.g), primary spermatocytes (sp.c.1), secondary spermatocytes (sp.c.2), spermatids and sperms (sp). Leydig cells (Ic) are noticed in the interstitial connective tissue.

Histological examination of testicular sections from the control group showed that the testicular tissue was formed of closely packed seminiferous tubules with regular intact basement membranes, patent lumina containing spermatozoa, and interstitial tissue among the tubules. Each tubule was lined by germinal epithelium formed of spermatogenic cells at different stages of development (spermatogonia, spermatocytes, spermatids, and spermatozoa) that were arranged from the base to the lumen of the tubules and Sertoli cells with their large pale nuclei was noticed in between. Spermatids were recognized by their darkly stained rounded nuclei and their position towards the lumen, while spermatozoa appeared elongated in shape with a pointed end. The flagella of mature sperms were seen filling the lumens of the seminiferous tubules. The tubules were separated from each other by loose interstitial connective tissue containing the Leydig cells which appeared with vesicular nuclei. A single layer of flat myoid cells resting on a consistent basement membrane was noticed in Figures 4A, B. Histological examination of the testicular sections from the diabetic group revealed that the testicular tissues were affected with variable degrees. Some seminiferous tubules appeared with irregular outlines and disorganized, detached, and shrunken germinal epithelial cells with pyknotic nuclei within the lumen. There were empty spaces between germ cells and decreased sperm flagella in the lumen of the tubules. Other tubules appeared depleted of most spermatogenic cells. Interstitium had congested blood vessels with homogenous vacuolated eosinophilic material (exudate, interstitial edema). The basement membrane was thin and irregular in Figures 4C–F. Histological examination of the testicular sections from the diabetic + liraglutide group revealed marked improvement in testicular histological features which was induced by liraglutide administration. Most seminiferous tubules showed restoration of their normal architecture with healthy-looking spermatogenic lineages, as the germinal epithelial cells were normally arranged (spermatogonia, spermatocytes, spermatids, and spermatozoa). Most tubular lumina contained aggregation of sperms. There was a nearly normal width of the interstitium in between the tubules. Little interstitial fluid exudate with few vacuoles was still noticed between some tubules in Figures 4G, H.

### 3.5 Immunohistochemical staining

**FIGURE 5:**
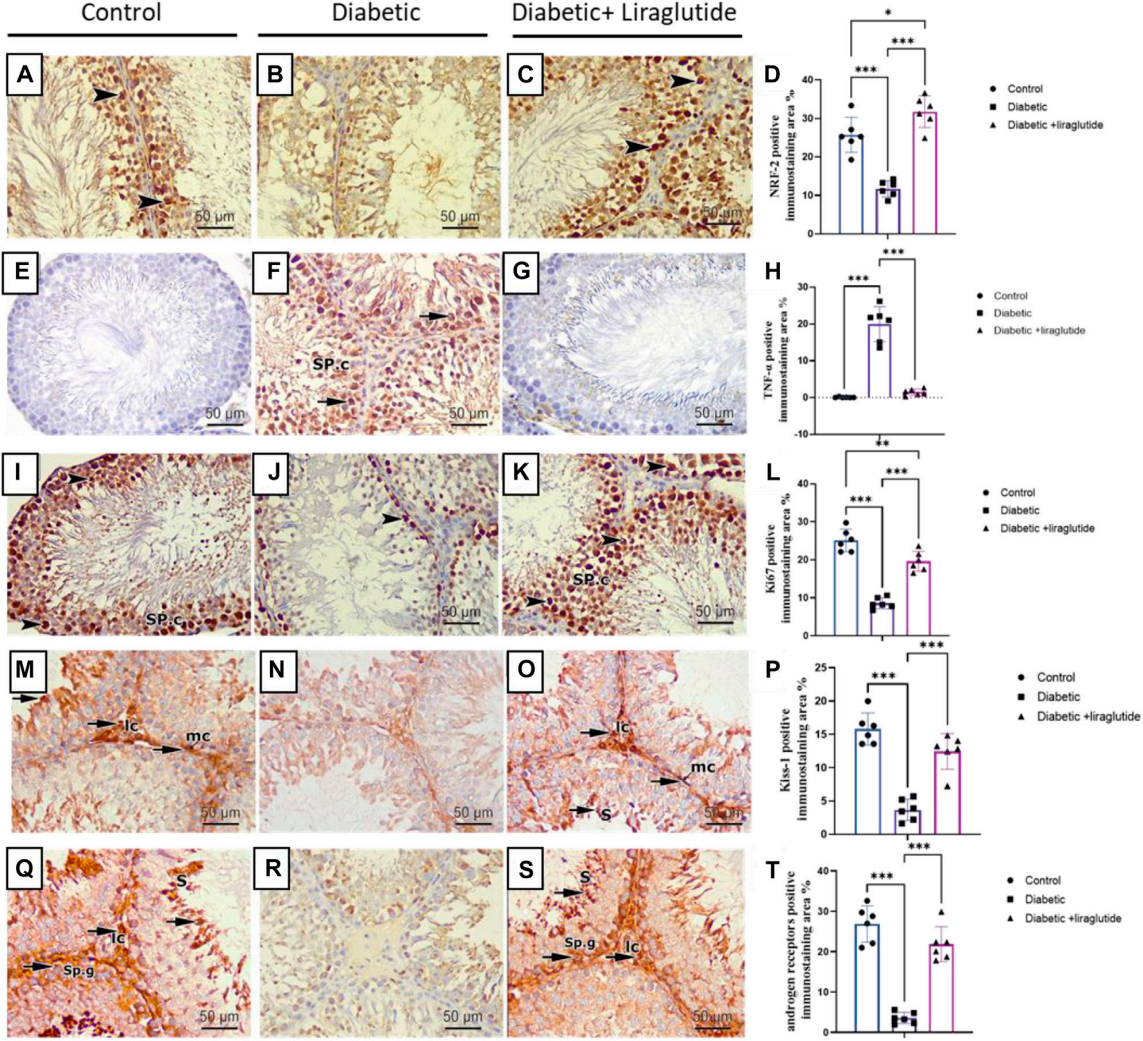
Immunohistochemical staining of testicular tissues of control, STZ rats, and STZ rats treated with liraglutide **(A–T)**. **(A)** Testicular immunohistochemical stained sections of Nrf2 in control groups Scale bar = 200 μm, x100, **(B)** Testicular immunohistochemical stained sections of Nrf2 in Diabetic group Scale bar = 200 μm, x100, **(C)** Testicular immunohistochemical stained sections of Nrf2 in Diabetic + liraglutide group Scale bar = 200 μm, x100, **(D)** Immunostaining intensity of testicular Nrf2 (% area), **(E)** Testicular immunohistochemical stained sections of TNFα in control groups Scale bar = 200 μm, x100, **(F)** Testicular immunohistochemical stained sections of TNFα in Diabetic group Scale bar = 200 μm, x100, **(G)** Testicular immunohistochemical stained sections of TNFα in Diabetic + liraglutide group Scale bar = 200 μm, x100, **(H)** Immunostaining intensity of testicular TNFα (% area), **(I)** Testicular immunohistochemical stained sections of Ki67 in control groups Scale bar = 200 μm, x100, **(J)** Testicular immunohistochemical stained sections of Ki67 in Diabetic group Scale bar = 200 μm, x100, **(K)** Testicular immunohistochemical stained sections of Ki67 in Diabetic + liraglutide group Scale bar = 200 μm, x100, **(L)** Immunostaining intensity of testicular Ki67 (% area), **(M)** Testicular immunohistochemical stained sections of Kisspeptin in control groups Scale bar = 200 μm, x100, **(N)** Testicular immunohistochemical stained sections of Kisspeptin in Diabetic group Scale bar = 200 μm, x100, **(O)** Testicular immunohistochemical stained sections of Kisspeptin in Diabetic + liraglutide group Scale bar = 200 μm, x100, **(P)** Immunostaining intensity of testicular Kisspeptin (% area), **(Q)** Testicular immunohistochemical stained sections of androgen receptors in control groups Scale bar = 200 μm, x100, **(R)** Testicular immunohistochemical stained sections of androgen receptors in Diabetic group Scale bar = 200 μm, x100, **(S)** Testicular immunohistochemical stained sections of androgen receptors in Diabetic + liraglutide group Scale bar = 200 μm, x100, **(T)** Immunostaining intensity of testicular androgen receptors (% area), Data are expressed as means ± SEM. *, **, *** indicate significant difference (*p* < 0.05, *p* < 0.01, and *p* < 0.001).

Immunohistochemical staining for immunoreactivity of the Nrf2 marker (which regulates the antioxidant defense mechanism of the cells against oxidative stress) revealed intense dark brown positive immunoreaction in the seminiferous tubular cells nuclei of the control group, while the diabetic group showed a scarce weak brown reaction in the tubular nuclei. In contrast, the administration of liraglutide in the last group induced upregulation of immunoexpression for Nrf2 in comparison to the diabetic group in Figures 5A–D. A strong positive brown cytoplasmic immunostaining for TNFα pro-inflammatory marker was noticed in the spermatogenic cells of the diabetic group. While negative cytoplasmic immunoreactivity was noticed in both control and diabetic + liraglutide groups in Figures 5E–H. Immunohistochemical staining for immunoreactivity of Ki67 proliferative marker revealed strong brown positive nuclear immunoreactivity in the spermatogenic cells lining the tubules of the control group, while the diabetic group showed a marked reduction in the immunoreactivity in comparison to the control group. A marked increase in the positive nuclear immunoreactivity was induced by liraglutide administration in the last group in Figures 5I–L. Expression and distribution patterns of both Kisspeptin and Androgen receptors in rat testicular tissues showed nearly similar results in the different studied groups. For control and diabetic + liraglutide groups, strong Kisspeptin and Androgen receptors immunoexpression were observed mainly in the Leydig cells of the interstitial testicular tissue, myoid cells, and spermatids but hardly detected in the remaining cells lining the seminiferous tubules. In contrast, the diabetic group showed marked attenuation of Kisspeptin and Androgen receptors expression that was reflected as negative immune stained cells in Figures 5M–T.

### 3.6 Hypothalamic and pituitary expression of GLP-1/leptin/kiss1/GnRH pathway

**FIGURE 6:**
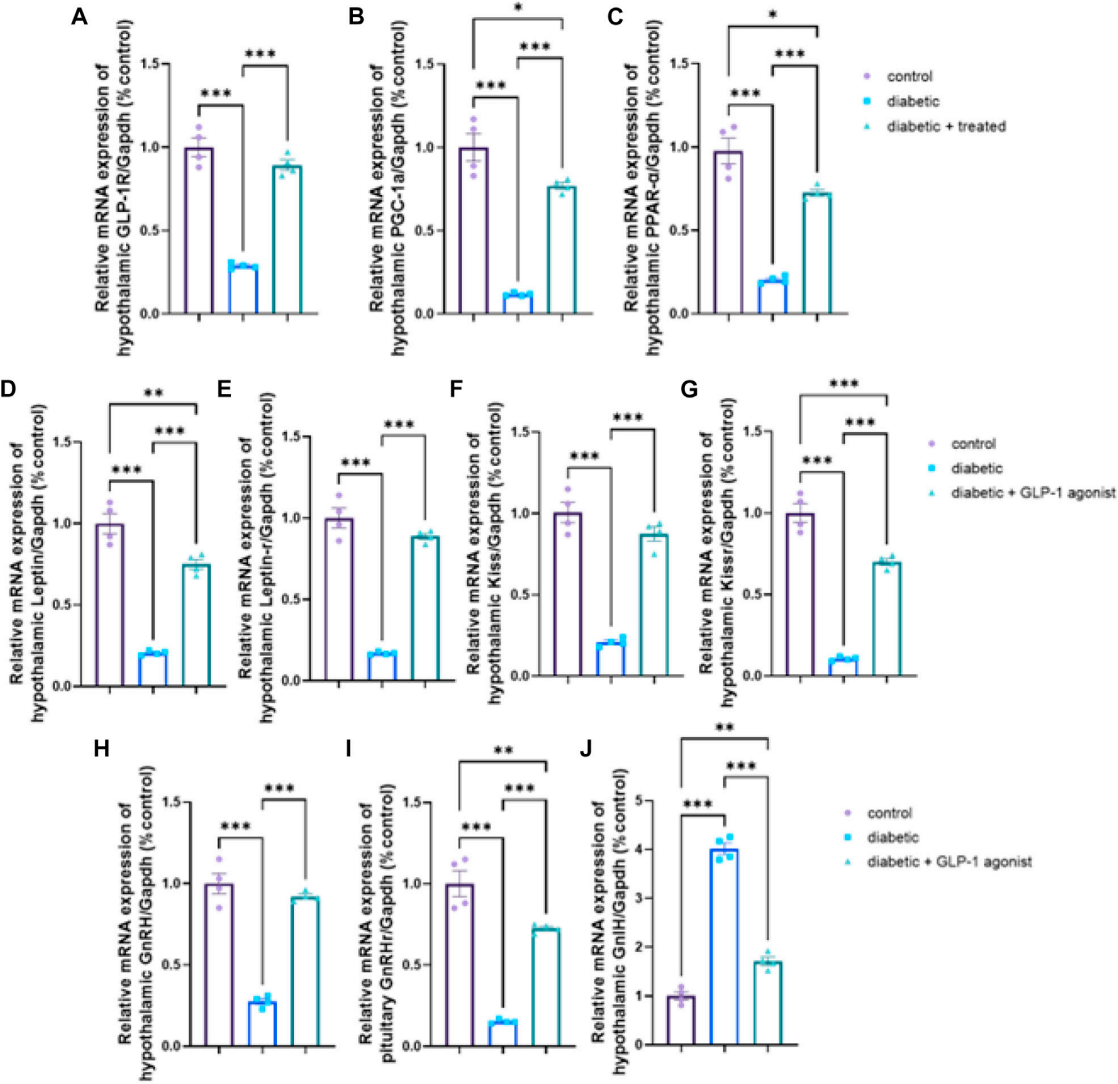
Effect of liraglutide on Hypothalamic and Pituitary expression of GLP-1/leptin/kiss1/GnRH pathway **(A–J)**. **(A)** GLP-1R, **(B)** PGC-1α, **(C)** PPAR-α, **(D)** leptin, **(E)** leptin R, **(F)** kiss, **(G)** kiss1R, **(H)** GnRH, **(I)** GnRHr, and **(J)** GnIH. Data are expressed as means ± SEM. *, **, *** indicate significant difference (*p* < 0.05, *p* < 0.01, and *p* < 0.001).

A significant downregulation in relative hypothalamic gene expression of GLP-1R, PPAR-α, PGC-1α, kiss, kiss1R, leptin, leptin R, GnRH, and upregulation of GnIH expression, as well as a significant downregulation in the pituitary mRNA expression of GnRHr, were found in diabetic group *versus* control (*p* < 0.0001) in Figures 6A–J. In group III, liraglutide treatment induced a significant upregulation in mRNA expression of hypothalamic GLP-1R, PPAR-α, PGC-1α, kiss1, kiss1R, leptin, leptin R, GnRH, and pituitary GnRH R as well as a significant downregulation in mRNA expression of hypothalamic GnIH when compared with expression levels of the diabetic group in Figures 6A–J.

### 3.7 Testicular expression of GLP-1/kiss1 and steroidogenesis pathway

**FIGURE 7:**
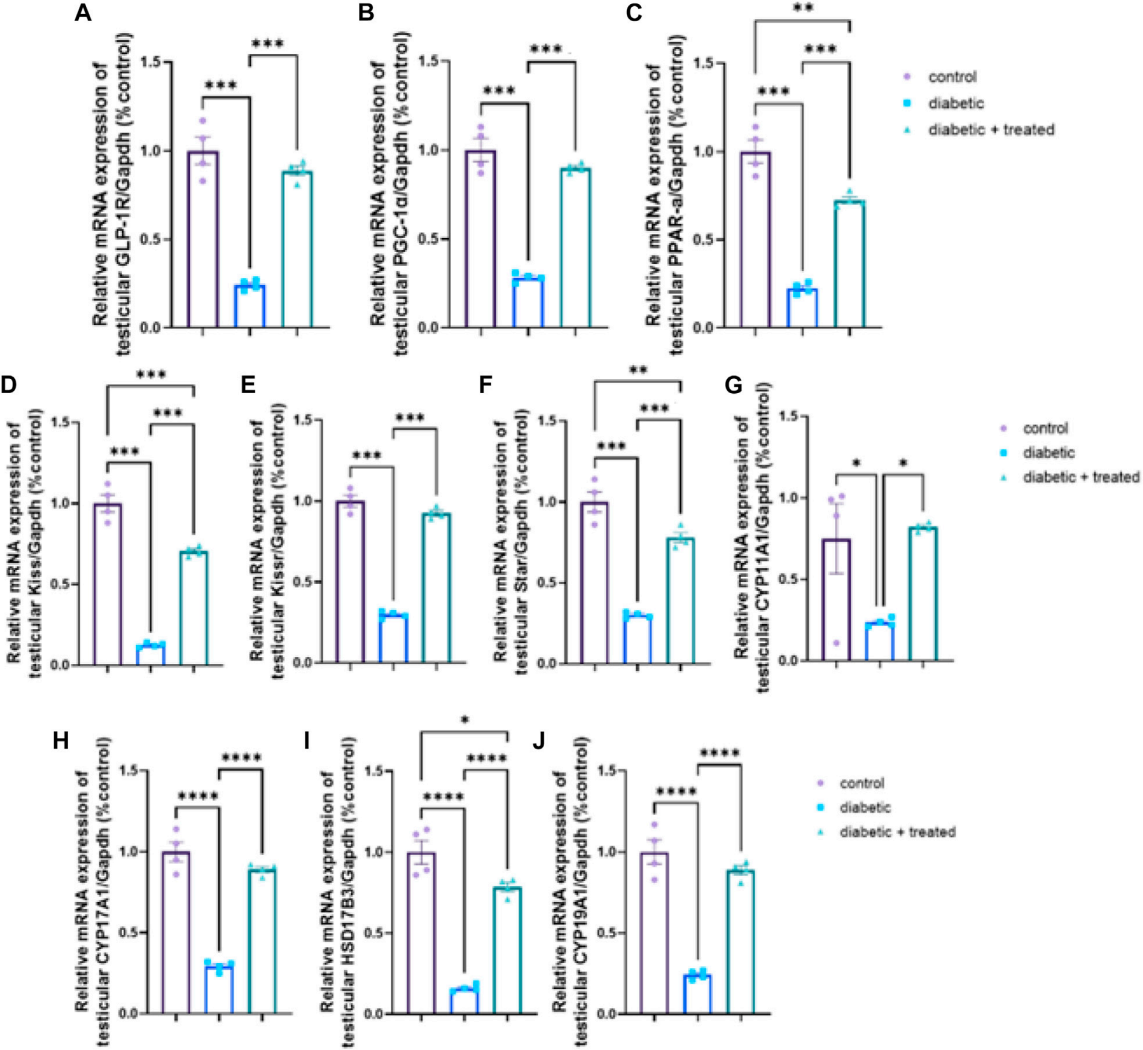
Effect of liraglutide on Testicular expression of GLP-1/kiss1 and steroidogenesis pathway **(A–J)**. **(A)** GLP-1R, **(B)** PGC-1α, **(C)** PPAR-α, **(D)** kiss1, **(E)** kiss1R, **(F)** STAR, **(G)** CYP11A1, **(H)** CYP17A1, **(I)** HSD17B3, and **(J)** CYP19A1. Data are expressed as means ± SEM. *, **, *** indicate significant difference (*p* < 0.05, *p* < 0.01, and *p* < 0.001).

The diabetic group showed significant downregulation in mRNA expression levels of testicular GLP-1R, PGC-1α, PPARα, kiss1, kiss1R, STAR, CYP17A1, HSD17B3, CYP19A1, and CYP11A1 compared to the control group in Figures 7A–J. Liraglutide administration in group III significantly upregulated their mRNA expression (*p* < 0.0001) compared to the diabetic group in Figures 7A–J.

### 3.8 Testicular expression of TGF- β/Smad pathway

**FIGURE 8:**
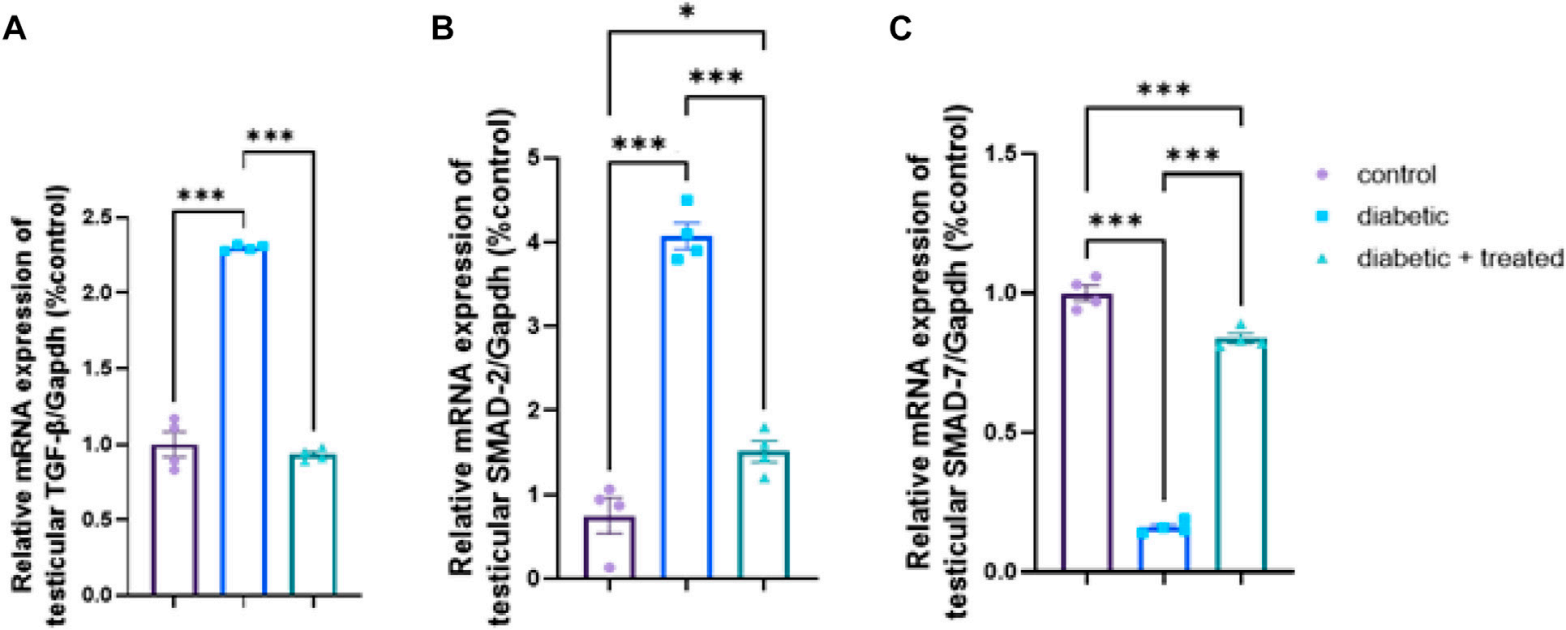
Effect of liraglutide on Testicular expression of TGF- β/Smad pathway **(A–C)**. **(A)** TGF- β, **(B)** Smad2, and **(C)** Smad7. Data are expressed as means ± SEM. *, **, ***indicate significant difference (*p* < 0.05, *p* < 0.01, and *p* < 0.001).

Compared to the control group, significant upregulation in mRNA expression of TGF- β, and Smad2 and significant downregulation of Smad7 were found in the diabetic group in Figures 8A–C. In group III, liraglutide administration significantly downregulated the mRNA expression of TGF- β, and Smad2 expression levels and upregulated Smad7 compared to the diabetic group in Figures 8A–C.

## 4 Discussion

Diabetes mellitus, a chronic metabolic disorder, is characterized by hyperglycemia due to defects in insulin secretion, insulin action, or both. Diabetes affects millions of people worldwide and is associated with numerous complications, including cardiovascular disease, neuropathy, retinopathy, and nephropathy. One of the lesser-known complications of diabetes is testicular dysfunction, which can lead to infertility and other reproductive problems. Diabetes-induced male testicular dysfunction is caused by several factors, including oxidative stress, inflammation, apoptosis, and hormonal imbalances (He et al., 2021). Diabetic-induced male testicular dysfunction is a complex disorder that requires a thorough understanding of the molecular mechanisms involved in the pathogenesis of the disease. In recent years, several therapeutic interventions have been explored in rats to target different molecular pathways involved in diabetic-induced male testicular dysfunction. A marked structural and functional impairment of reproductive function in diabetic rats has been reported (Mao et al., 2018; Barsiah et al., 2019; Öztaş et al., 2019). In the testis, a significant disruption of seminiferous tubular architecture with decreased spermatic content and atrophied germinal epithelium and interstitial tissue was noticed (Mao et al., 2018; Barsiah et al., 2019; Öztaş et al., 2019). These changes are secondary to chronic metabolic complications of diabetes including chronic hyperglycemia, insulin resistance, and dyslipidemia which initiate an inflammatory response as demonstrated by positive testicular immune staining for TNFα and a significant increase in serum TNFα and IL6 (Kwon and Pessin, 2013; Long et al., 2018; Barsiah et al., 2019). Oxidative stress is linked to reproductive disorders by augmenting insulin resistance through activation of the N-terminal kinase pathway (Yang et al., 2019), protein kinase C (Delarue and Magnan, 2007), and induction of several transcriptional factors (Sahin et al., 2019). Chronic hyperglycemia also upregulates the number of mitochondria and lipid peroxidation causing inhibition of antioxidant defense mechanisms (Song et al., 2019).

Liraglutide is a glucagon-like peptide-1 (GLP-1) receptor agonist used to treat type 2 diabetes (Abdel-Hakeem et al., 2023). The medication has been shown to have several health benefits beyond its blood sugar-lowering effects, including weight loss, cardiovascular protection, and potential effects on testicular function (Pourheydar et al., 2021). Administration of liraglutide in group III was able to decrease the damage in the reproductive system with significant improvement of testicular histopathologic findings and return to an almost normal structure. This improvement could be a result of the favorable effect of liraglutide on metabolic state indicated by a significant correction of glucose homeostatic parameters, lipid profile, and inflammatory and oxidative stress markers in concomitant with a significant reduction in BMI. Liraglutide administration has been associated with an increased immune expression of androgen receptors and the proliferative marker Ki67. Such changes favor the growth and regeneration of testicular tissue and can improve reproductive function as previously described (Giagulli et al., 2015; Jensterle et al., 2019).

The GLP-1-kisspeptin-GnRH signaling pathway is a key central molecular pathway involved in diabetic-induced male testicular dysfunction (Skorupskaite et al., 2014; Abdullah et al., 2022). GLP-1 is a hormone that is produced by the intestines in response to food intake, and it has been shown to have beneficial effects on glucose metabolism and insulin sensitivity. GLP-1 receptors are also present in the hypothalamus and pituitary gland, where they play a key role in the regulation of reproductive function. Kisspeptin is a neuropeptide that is produced by neurons in the hypothalamus, and it plays a key role in the regulation of gonadotropin-releasing hormone (GnRH) secretion. GnRH is a hormone that is produced by the hypothalamus and stimulates the pituitary gland to release follicle-stimulating hormone (FSH) and luteinizing hormone (LH), which are essential for the development and maturation of sperm. In diabetic rats, the GLP-1-kisspeptin-GnRH signaling pathway is disrupted, leading to impaired gonadotropin secretion and testicular dysfunction. In the diabetic group, the results of this study showed significant downregulation in the hypothalamic expression of GLP-1R, PPAR-α, PGC-1α, kiss, kiss1R, leptin, leptin R, GnRH, and pituitary GnRHR as well as upregulation of hypothalamic GnIH expression. Kisspeptin is essential for normal activity of the HPG axis and plays a vital role in initiating puberty (Baldauff and Witchel, 2017; Brown et al., 2017). Kisspeptin stimulates GnRH secretion and its deficiency leads to hypogonadotropic hypogonadism, while insulin deficiency and insulin resistance were found to decrease hypothalamic Kisspeptin expression with a subsequent decrease in GnRH secretion (Öztin et al., 2016). Moreover, Leptin was reported to regulate the expression of hypothalamic Kiss1 in male mice and about 40% of Kiss1 neurons in the arcuate nucleus contain functional leptin receptors (Brothers et al., 2010). Taken together, diabetic insult on reproductive function is not mediated only by metabolic disturbance but also by inversely affecting the HPG axis.

GLP-1 is involved in the regulation of HPG function, as it modulates the activity of GnRH neurons in the hypothalamus (Beak et al., 1998; Oride et al., 2017). In neuronal cell lines, GLP-1 was able to increase LH-releasing hormone (Beak et al., 1998), while in animal studies, GLP-1 induced GnRH release from hypothalamic neurons (Farkas et al., 2016) and increased the expression of Kiss-1 gene and secretion of kisspeptin (Oride et al., 2017). These data are in line with the results of this study as in liraglutide treated group, a significant upregulation of hypothalamic GLP-1R, PPAR-α, PGC-1α, kiss1, kiss1R, leptin, leptin R, GnRH, and the pituitary GnRH R as well as a significant downregulation of GnIH were noticed. These effects increased pituitary gonadotropic hormones and eventually testicular hormones which were indicated by the significant increase in serum LH, FSH, and testosterone levels. Similar results were obtained from some human (Giagulli et al., 2015; Jensterle et al., 2019) and animal studies (Ahangarpour et al., 2014; Zhang et al., 2015). Opposite to our findings, some human studies reported a reduction in testosterone pulse with extended pulse duration after Liraglutide infusion (Jeibmann et al., 2005), while others reported no effect on testosterone or LH pulse (Izzi-Engbeaya et al., 2020). The difference might be attributed to the mode of administration (acute *versus* chronic) or study groups (healthy *versus* diseased) or even species differences may be a cause of such discrepancy.

The observed effects of liraglutide on reproductive function can be either secondary to its effects on body weight and metabolic health, or a direct effect of GLP-1 on the reproductive system. Both remain valid pathways. Some case reports showed GLP-1 treatment damaged reproduction function in one patient (Fontoura et al., 2014). Recently, La Vignera et al. reported preliminary results of a clinical trial of 110 men with metabolic hypogonadism, and showed liraglutide treatment improved male reproductive functions as indicated by sperm parameters, erectile functions, and serum levels of total testosterone, sex hormone-binding globulin, and gonadotropin (La Vignera et al., 2023). However, it is also worth noting that GLP-1 receptors are present in the hypothalamus, and there is evidence to suggest that GLP-1 may play a role in regulating appetite and energy balance through effects on this brain region. Additionally, a recent study on testicular dysfunction in a testicular ischemia-reperfusion rat model reported that early intervention of Semaglutide (another GLP-1 agonist) attenuated testicular dysfunction by targeting the GLP-1–PPAR-α–Kisspeptin–Steroidogenesis signaling pathway where injecting exendin, a GLP1-R antagonist, before semaglutide abolished all the documented improvements [1]. Overall, the mechanisms underlying the effects of liraglutide on reproductive function are likely complex and multifactorial, and further research is needed to fully understand them. As for HPG, the testicular GLP-1/kiss1signalling pathway (GLP-1R, PGC-1α, PPARα, kiss1, and kiss1R) was downregulated in the diabetic group and significantly upregulated after liraglutide treatment in group III which presumably improved testicular energy balance and directly boosted spermatogenesis and steroidogenesis, an effect that was evident in our results in by the significant upregulation in the expression of steroidogenesis related genes (STAR, CYP11A1, CYP17A1, HSD17B3, and CYP19A1). TGF- β prominently regulates testicular function including endocrine function, spermatogenesis, growth, and development of the testis (Itman et al., 2006; Kabel, 2018). However, Lebrin et al. showed that TGF- β has bi-functional effects as it can induce or inhibit cell proliferation (Lebrin et al., 2005). Activation of the TGF- β pathway was reported to induce fibrotic and apoptotic changes in the testis and hence impair reproductive function (Shiraishi et al., 2003; Monsivais et al., 2017). Studying the testicular expression of TGF- β/Smad signaling pathway demonstrated a significant upregulation in mRNA expression of TGF-β and Smad2 and a significant downregulation in mRNA expression of Smad7 in the diabetic group while liraglutide treatment in group III reversed these changes; downregulating TGF-β and Smad2 but upregulating Smad7. TGF-β signal transduction pathway highly depends on Smad proteins (Blobe et al., 2000). TGF-β phosphorylates type I receptor Smad2, which after interaction with Smad3 and Smad4 induces transcription of fibrotic-related genes (ten Dijke and Hill, 2004; Tzavlaki and Moustakas, 2020) leading to fibrotic and degenerative changes in the testis. Smad 7 is one of the inhibitory Smads which interfere with the action of Smad2 (Hayashi et al., 1997; Imamura et al., 1997), so its downregulation in the diabetic group aggravates the action of TGF-β causing more testicular damage. Consistent with this study’s results, Zheng et al., reported upregulation of TGF- β and Smad2 expression in diabetic rats resulting in testicular interstitial fibrosis (Zheng et al., 2021) while Liu et al., found testicular fibrosis in diabetic rats associated with upregulated TGF-β which was reversed by vitamin D3 (Liu et al., 2019). Moreover, liraglutide treatment was reported to decrease TGF- β and immune cell infiltration in pulmonary fibrosis (Gou et al., 2014; Zhao et al., 2014) and diabetic nephropathy (Chen et al., 2018).

The reproductive action of TGF- β extends to steroidogenesis as it was reported to downregulate the expression of LH and hCG receptors and in turn decrease hCG-induced testosterone production in Leydig cells and it was also reported to decrease steroidogenic genes expression as StAR and P450c17 (Ingman and Robertson, 2007) as the upregulation of TGF- β/Smad2 in diabetic group was associated with downregulation of steroidogenic genes expression (STAR, CYP11A1, CYP17A1, HSD17B3, and CYP19A1) and decrease in total serum testosterone, all of which were improved in group III after liraglutide treatment.

## 5 Conclusion

The central and peripheral molecular pathways involved in diabetic-induced male testicular dysfunction in rats are complex and multifactorial and involve several different cellular and molecular mechanisms. Understanding these pathways is essential for the development of effective treatment strategies for this condition. Type II diabetes can markedly impair gonadal structure and function as a result of metabolic complications, chronic inflammation, oxidative stress, and inhibiting testicular steroidogenesis directly and indirectly by suppressing the HPG axis. Administration of GLP-1RA, liraglutide can be beneficial in preventing and/or managing Type II diabetes-associated reproductive complications, significantly reversing the deleterious diabetic impacts and improving reproductive function through GLP-1/kiss1/leptin/GnRH and TGF-β/Smad pathways indicating additional benefits for GLP-1RAs other than dealing with diabetes.

## Data Availability

The original contributions presented in the study are included in the article/Supplementary Material, further inquiries can be directed to the corresponding authors.
