# Supplementary material for: Liraglutide ameliorates diabetic-induced testicular dysfunction in male rats: role of GLP-1/Kiss1/GnRH and TGF-β/Smad signaling pathways
Source: Front Pharmacol. 2023 Jul 11;14:1224985. doi: 10.3389/fphar.2023.1224985 (PMC10367011; doi:10.3389/fphar.2023.1224985)
Supplement: Supplementary file 1 [file DataSheet1.pdf]

Liraglutide Ameliorates Diabetic-Induced Testicular Dysfunction in Male Rats: Role of GLP-1/Kiss1/GnRH and TGF-β/Smad Signaling Pathways

Maha Abdelhamid Fathy [1], Amira Ebrahim Alsemeh [2], Marwa A. Habib [1], Hanim M. Abdel-nour [3], Doaa M. Hendawy[3], Asmaa Monir Eltaweel [4], Adel Abdelkhalek [5], Mona M Ahmed [6], Maha K. Desouky [7], Jinlian Hua [8], Liana Mihaela FERICEAN[9], Ioan BANATEAN-DUNEA [9,*], Ahmed Hamed Arisha [10,*], Tarek Khamis [11,*]

[1] Medical Physiology Department, Faculty of Medicine, Zagazig University, 44519 Zagazig, Egypt

[2] Human Anatomy and Embryology Department, Faculty of Medicine, Zagazig University 44519 Zagazig, Egypt

[3] Medical Biochemistry Department, Faculty of Medicine, Zagazig University, 44519 Zagazig, Egypt

[4] Basic medical science department (Anatomy and Embryology) - College of medicine-King Saud Abdulaziz University for Health Sciences - Kingdom of Saudi Arabia & Human Anatomy and Embryology Department, Faculty of Medicine, Zagazig University 44519 Zagazig, Egypt

[5] Faculty of Veterinary Medicine, Badr University in Cairo, Badr City, 11829, Egypt

[6] Department of Forensic Medicine and Toxicology, Faculty of Veterinary Medicine, Zagazig University, Zagazig, 44511 Zagazig, Egypt

[7] Department of Anatomy, Faculty of Medicine, Minia University, 61511 Minia, Egypt

[8] College of Veterinary Medicine/Shaanxi Centre of Stem Cells Engineering & Technology, Northwest Agriculture & Forestry University, Shaanxi, Yangling, China

[9] Department of Biology, Faculty of Agriculture, University of Life Sciences, King Mihai I" from Timisoara [ULST], Aradului St. 119, 300645 Timisoara, Romania; ioan_banatean@usab-tm.ro (I.B-D)

[10] Department of Animal Physiology and Biochemistry, Faculty of Veterinary Medicine, Badr University in Cairo, Badr City, Egypt and Department of Physiology, and Laboratory of Biotechnology, Faculty of Veterinary Medicine, Zagazig University, Zagazig 44511, Egypt; vetahmedhamed@zu.edu.eg

[11] Department of Pharmacology and Laboratory of Biotechnology, Faculty of Veterinary Medicine, Zagazig University, 44519 Zagazig, Egypt; t.khamis@vet.zu.edu.eg

*** Correspondence:**
ioan_banatean@usab-tm.ro (I.B-D.)
vetahmedhamed@zu.edu.eg (A.H.A.)
t.khamis@vet.zu.edu.eg (T.K.)

pit & hypo

kiss

control	diabetic	diabetic + treated
1.15	0.21	0.89
0.87	0.18	0.75
1.06	0.24	0.94
0.94	0.2	0.92

kiss-r

control	diabetic	diabetic + treated
1.07	0.12	0.65
0.93	0.11	0.72
0.88	0.09	0.74
1.12	0.1	0.69

GnRH

control	diabetic	diabetic + treated
1.03	0.31	0.96
0.97	0.29	0.89
0.85	0.27	0.91
1.15	0.23	0.92

GnRHr

control	diabetic	diabetic + treated
0.85	0.15	0.75
1.15	0.17	0.69
0.88	0.14	0.74
1.12	0.15	0.72

leptin

control	diabetic	diabetic + treated
0.87	0.2	0.68
1.13	0.19	0.72
0.92	0.22	0.81
1.08	0.21	0.79

leptinr

control	diabetic	diabetic + treated

0.96	0.17	0.88
1.04	0.16	0.84
1.14	0.164	0.92
0.86	0.18	0.91

GLP-1R

control	diabetic	diabetic + treated
0.88	0.29	0.97
1.12	0.28	0.91
0.94	0.31	0.86
1.06	0.27	0.83

PGC-1a

control	diabetic	diabetic + treated
0.89	0.11	0.79
1.11	0.11	0.72
0.83	0.13	0.81
1.17	0.12	0.76

ppar-a

control	diabetic	diabetic + treated
1.09	0.23	0.69
0.81	0.18	0.72
1.12	0.19	0.78
0.88	0.21	0.71

GnIH

control	diabetic	diabetic + treated
1.07	3.79	1.92
0.93	4.26	1.67
1.19	4.17	1.52
0.81	3.85	1.73

testis

kiss

control	diabetic	diabetic + treated
1.05	0.14	0.74
0.95	0.11	0.67
1.12	0.13	0.69
0.88	0.12	0.72

kiss-r

control	diabetic	diabetic + treated
1.03	0.32	0.97
0.97	0.29	0.91
1.08	0.31	0.89
0.92	0.27	0.93

CYP11A1

control	diabetic	diabetic + treated
1.01	0.22	0.83
0.99	0.27	0.81
0.89	0.21	0.79
0.11	0.24	0.85

Star

control	diabetic	diabetic + treated
1.06	0.31	0.75
0.94	0.29	0.86
1.14	0.32	0.72
0.86	0.28	0.79

CYP17A1

control	diabetic	diabetic + treated
1.05	0.25	0.91
0.95	0.29	0.89
0.86	0.31	0.92
1.14	0.32	0.84

HSD17B3

control	diabetic	diabetic + treated
1.14	0.19	0.78
0.86	0.15	0.71
0.89	0.16	0.82
1.11	0.14	0.83

CYP19A1

control	diabetic	diabetic + treated
1.07	0.26	0.89
0.93	0.23	0.81
1.17	0.27	0.91
0.83	0.21	0.94

GLP-1R

control	diabetic	diabetic + treated
1.07	0.26	0.89
0.93	0.23	0.81
1.17	0.27	0.91
0.83	0.21	0.94

PGC-1a

control	diabetic	diabetic + treated
1.08	0.25	0.91
0.92	0.29	0.89
0.87	0.28	0.87
1.13	0.31	0.93

PPAR-a

control	diabetic	diabetic + treated
1.07	0.19	0.69
0.93	0.21	0.72
1.14	0.26	0.78
0.86	0.24	0.71

TGF-b

control	diabetic	diabetic + treated
0.83	0.28	0.89
1.17	0.31	0.91
0.89	0.29	0.95
1.11	0.32	0.98

SMAD-2

control	diabetic	diabetic + treated
0.87	4.5	1.2
0.13	3.9	1.8
0.94	4.1	1.6
1.06	3.8	1.45

SMAD-7

control	diabetic	diabetic + treated
0.94	0.19	0.89
1.06	0.14	0.83
0.97	0.16	0.81
1.03	0.15	0.82
